# An implementation evaluation of the physical activity counseling for in-patients with major depressive disorder (PACINPAT) intervention: a randomized controlled trial

**DOI:** 10.1186/s12888-023-04834-5

**Published:** 2023-05-04

**Authors:** Robyn Cody, Jan-Niklas Kreppke, Xenia Fischer, Oliver Faude, Johannes Beck, Serge Brand, Martin Hatzinger, Christian Imboden, Nadine Kügerl, Undine E. Lang, Sarah Mans, Reto Maurer, Thorsten Mikoteit, Anja Oswald, Lilja-Sophie Rhodius, Nina Schweinfurth, Laura Wechsler, Markus Gerber

**Affiliations:** 1 Department for Sport, Exercise and Health, University of Basel, Grosse Allee 6, 4052 Basel, Switzerland; 2 Psychiatric Clinic Sonnenhalde, Riehen, Switzerland; 3 Adult Psychiatric Clinics (UPKE), University of Basel, Basel, Switzerland; 4 Sleep Disorders Research Center, Kermanshah University of Medical Sciences, Kermanshah, 6719851115 Iran; 5 Substance Use Prevention Research Center and Sleep Disorder Research Center, Kermanshah, University of Medical Sciences (KUMS), Kermanshah, 6715847141 Iran; 6 School of Medicine, Tehran University of Medical Sciences (TUMS), Tehran, Iran; 7 Psychiatric Services Solothurn, Solothurn, Switzerland; 8 Private Clinic Wyss, Münchenbuchsee, Switzerland

**Keywords:** Intervention implementation, Process evaluation, Physical activity counseling, Depression

## Abstract

**Background:**

The physical activity counseling for in-patients with major depression (PACINPAT) randomized controlled trial was launched to tackle physical inactivity for in-patients with major depressive disorder. Evidence shows that despite potential treatment effects, physical inactivity is prevalent in this population. To contribute to the assessment of how this in-person and remote, theory-based, individually tailored intervention was designed, received and effected behavior, the aim of this study was to evaluate its implementation.

**Methods:**

This implementation evaluation was conducted within a multi-center randomized controlled trial according to the Process Evaluation Framework by the Medical Research Council including the analysis of reach, dose, fidelity and adaptation. Data were collected from the implementers and the participants randomized to the intervention group of the trial.

**Results:**

The study sample comprised 95 physically inactive in-patients (mean age: 42 years, 53% women) with diagnosed major depressive disorder. The intervention reached the intended population (*N* = 95 in-patients enrolled in the study). The intervention dose varied between early dropouts (counseling sessions, *M* = 1.67) and study completers with some participants receiving a low dose (counseling sessions, *M* = 10.05) and high dose (counseling sessions, *M* = 25.37). Differences in the attendance groups were recognizable in the first two counseling sessions (duration of counseling session about 45 min in early dropouts versus 60 min for study completers). Fidelity of the in-person counseling content was partly achieved and adapted, whereas that of the remote counseling content was well achieved. Participants (86% at follow up) reported satisfaction with the implementers of the intervention. Adaptations were made to content, delivery mode and dose.

**Conclusion:**

The PACINPAT trial was implemented in the intended population, in varying doses and with adaptations made to in-person counseling content and remote counseling dose. These findings are key to understanding outcome analyses within the PACINPAT trial, further developing interventions and contributing to implementation research among in-patients with depressive disorders.

**Trial registration:**

ISRCTN, ISRCTN10469580, registered on 3^rd^ September 2018.

## Background

Worldwide, 27.5% of adults are not sufficiently physically active [1]. It is estimated that physical inactivity causes 9% of premature mortality, 6% of the burden of coronary heart disease, 7% of type 2 diabetes, 10% of breast cancer and 10% of colon cancer globally [2]. Additionally, it has been shown that people with higher levels of physical activity have lower odds of developing depression (adjusted odds ratio = 0.83, 95% CI 0.79 to 0.88) [3]. In turn, according to meta-analytic data, people with depression tend to be less physically active than peers without depression (standard mean difference = -0.25, 95% CI -0.03 to 0.15) and over half (67%) do not meet physical activity recommendations [4]. It would appear to be worthwhile to promote physical activity among people with major depressive disorders because according to meta-analytic data an average of 45 min of moderate-intensity physical activity, three times per week for approximately 9 weeks leads to a significantly large antidepressant effect (*g* = -0.79, 95% CI -1.01 to -0.57) [5]. This evidence is further corroborated by a meta-analysis of randomized controlled trials showing that exercise interventions have a large effect (standard mean difference = -0.95, 95% CI -1.18 to -0.71) on depressive symptoms [6]. Thus underlining the need of interventions to facilitate a more physically active lifestyle among people with major depressive disorders.

Physical activity is defined as any bodily movement resulting from skeletal muscles, which in turn results in energy expenditure [7]. Hence, there is a wide range of possibilities to be physically active: occupationally, doing sports, conditioning, in a household, or other activities [7]. Physical activity counseling is an educational, client-centered and goal-oriented approach whereby the aim is to achieve lasting behavior change through a cooperative relationship and empowerment of the individual undergoing said change [8].

Physical activity counseling has been effective in changing physical activity behavior in physically inactive adults (increases of 32 min/week, 95% CI 0.1 to 63, at intervention follow up) [9]. Similarly, in people with depression, facilitated physical activity counseling has led to increased physical activity levels (adjusted odds ratio 2.27, 95% CI 1.32 to 3.89) in out-patients [10]. To investigate the efficacy of an in-person and remote, theory-based, individually tailored physical activity counseling intervention in in-patients with depression, the physical activity counseling for in-patients with major depression (PACINPAT) multi-center randomized controlled trial (RCT) was launched. The novelty of this trial is that the intervention content is theory-based yet personalized to the recipient, it takes place in-person as well as remotely for 1 year to facilitate maintenance, it is delivered in in-patient care and the primary endpoint is objectively measured physical activity (accelerometer). The trial protocol has been published previously [11].

**Fig. 1:**
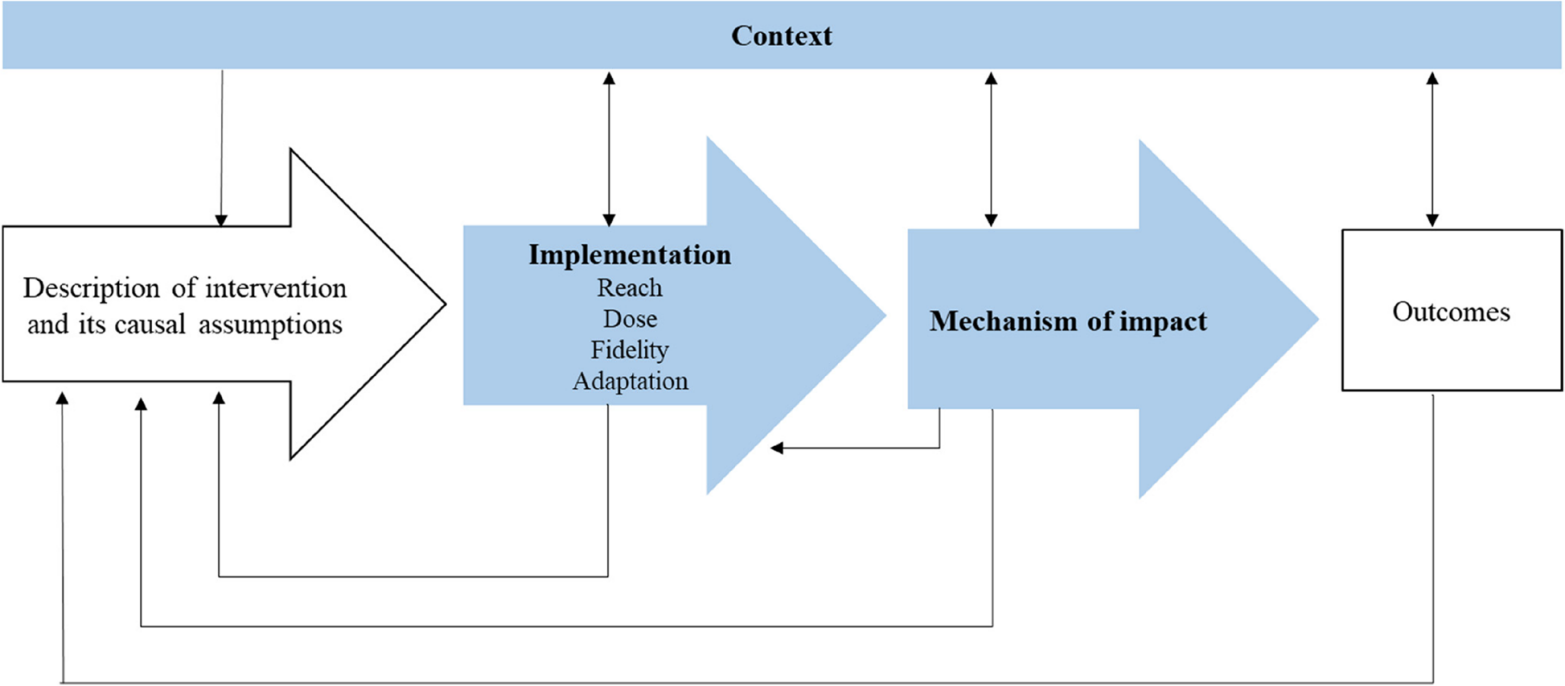
Process evaluation according to Moore et al. [14]

The impact of such a physical activity counseling intervention depends not only on its efficacy, as assessed within the RCT, but also its reach, adoption, implementation and maintenance [12]. Hence, additional assessments are required to adequately evaluate a complex intervention [13]. Along these lines, the Medical Research Council (MRC) provides a framework for the process evaluation of complex interventions as depicted in Fig. 1 [14]. The framework describes three components to be considered, namely implementation (what is implemented and how?), mechanism of impact (how does the delivered intervention produce change?) and context (how does context affect implementation and outcomes?). The evaluation of intervention implementation in turn consists of four components; reach (target audience), dose (of delivery and receipt), fidelity (delivered as intended) and adaptations (modifications for contextual fit).

In the case of the PACINPAT trial, the intervention and its causal assumptions have been described in the study protocol [11]. The mechanism of impact was assessed in a nested qualitative study on the participants’ experience of the intervention [15]. Upon completion of the trial, outcomes with respect to physical activity levels and secondary outcomes as defined in the study protocol will be published. According to the MRC framework, the context shaping the causal assumptions of the intervention, as well as its implementation, mechanism of impact and outcomes is to be considered in each step of the evaluation process.

Hence, the aim of the present study is threefold: First, to evaluate the implementation of the intervention by addressing the following questions: (i) did the intervention reach the targeted audience? (ii) how many of the planned counseling sessions took place and how long was their duration (dose)? (iii) was the content delivered as planned? and (iv) what, if any, adaptations were made to the intervention? Second, to analyze whether the duration and content of the counseling sessions differed according to the intervention dose. And third, to present the recipients’ satisfaction with the intervention.

By establishing the actual dose of the intervention potential future implementation in practice in terms of remuneration of implementers can be informed, as the entire counseling duration (including preparation and debriefing time) will become visible.

This study will therefore complete the process evaluation of the PACINPAT trial and allow a deeper understanding of what may account for future observed effects of this physical activity intervention for people with major depression. The aim of the study is therefore to evaluate the implementation of the intervention within the PACINPAT multi-center randomized controlled trial based on quantitative data provided by the implementers and participants of the PACINPAT trial.

## Methods

### Setting and participants

The PACINPAT trial is being conducted in four Swiss psychiatric clinics (study cites in Basel, Riehen, Solothurn and Münchenbuchsee). The participants were screened by clinicians according to the following inclusion criteria: adult (18–65 years) women and men with episodic or recurrent moderate to severe depression according to the International Classification of Disease, 10^th^ Edition (ICD-10), who were physically insufficiently active upon admission to in-patient treatment (< 150 min of moderate-to-vigorous physical activity per week [16]). Once clinically screened, the clinicians referred the patient to a member of the study team who explained the study procedures, emphasizing the voluntary basis of participation and anonymity of data. Patients who decided to take part signed a written informed consent form and were randomized 1:1 with a permuted block randomization with the strata age, sex, and clinic into an intervention and control group. The participants were blind to group allocation while, given the nature of the intervention the implementers were not. Data used in this publication pertain only to the participants randomized into the intervention group.

### Development of the intervention

The intervention consisted of physical activity counseling which took place in-person and remotely including text messages and the use of a mobile phone application during one year. The delivery mode, timing, content and underpinning theory are shown in Table 1.

**Table 1:** Intervention design

	**Session**	**Delivery**	**Timing**	**Content**	**Theory**
Phase 1: in-person	1	In-person (2 h)	During in-patient treatment	- Introduction and organizational aspects of the intervention - Health goals - Physical activity ideas	MoVo
	2	In-person (1.5 h)	During in-patient treatment	- Physical activity plan (what, when, where and with whom) including check for suitability, practicability, precision and effectiveness - Instruction for self-monitoring of physical activity including introduction to mobile app as self-monitoring tool - Introduction to remote sessions - Appointment for next session (first telephone session)	MoVo
Phase 2: remote	3	Telephone (1 h)	2 weeks after discharge from in-patient treatment	- Physical activity barriers (internal and external) - Strategies (e.g., prioritizing physical activity, making an appointment with a friend, preparing required equipment) - Instruction to implement physical activity plan, self-monitoring and strategies - Introduction to text messages - Appointment for next session	MoVo
	4–28	Telephone (0.5 h)	Bi-weekly (26 in 12 months)	- Check implementation of physical activity plan, self-monitoring and strategies - Appropriate BCTs selected from the Anchor List - Intermittent goal setting and reviewing in sessions 4, 9, 14, 19, 24 and 28 (every 2 months) - Appointment for next session	BCW
		Text messages	Weekly (52 in 12 months)	- Feedback - Reminder - Information	BCW
		Mobile App		- Physical activity diary including setting of time, place, with whom, duration and intensity, including the option to confirm the activity took place - Profile to save anthropological and physical activity data - Notepad to summarize goals, ideas, barriers and strategies	

*h* hours, *App* mobile application, *BCT* Behavior Change Technique, *MoVo* Motivation Volition Model, *BCW* Behavior Change Wheel

The intervention was theory-based. The first three sessions were based on the Motivation-Volition (MoVo) Model developed by Fuchs and colleagues [17]. The model assumes that strengthening both motivational (self-efficacy and goal intention) and volitional (action planning and barrier management) constructs, behavior can be initiated and maintained through positive outcome experiences [17]. There is a corresponding intervention designed as a short physical activity counseling intervention to be delivered to in-patients [18], according to which the initial stages of the present intervention were designed. In the following stages, the intervention was based on the Behavior Change Wheel (BCW), which is a framework containing concepts from health behavior theories [19]. At the core of the wheel, behavior is explained by capability (physical and psychological), opportunity (environmental and social) and motivation (reflective and automatic). There are 93 Behavior Change Techniques (BCTs), defined according to a taxonomy (version 1), which can be implemented to target these determinants of behavior [20]. A BCT “Anchor List” was developed in a previous study [9], containing thirty BCTs. Ten of which, were effective for physical activity promotion according to evidence and were thus classified as main BCTs. The remaining twenty BCTs were explicitly or implicitly used in physical activity counseling according to evidence and were thus classified as secondary BCTs. This “Anchor List” was used for the present intervention (see Table 2).

**Table 2:** Behavior change techniques anchor list for physical activity

Taxonomy Version 1
**1.1. Goal setting (behavior)**
1.3. Goal setting (outcome)
**1.2. Problem solving**
**1.4. Action planning**
8.7. Graded tasks
**1.5. Review behavior goal(s)**
1.7. Review outcome goal(s)
1.6. Discrepancy between current behavior and goal
1.8. Behavioral contract
**2.2. Feedback on behavior**
2.7. Feedback on outcomes of behavior
**2.3. Self-monitoring of behavior**
2.4. Self-monitoring of outcomes of behavior
**3.1. Social support (unspecified)**
**4.1. Instruction on how to perform the behavior**
**5.1. Information about health consequences**
5.2. Salience of consequences
6.1. Demonstration of the behavior
6.2. Social comparison
7.1. Prompts/cues
8.1. Behavioral practice/rehearsal
**8.3. Habit formation**
9.2. Pros and cons
10.3. Non-specific reward
12.1. Restructuring the physical environment
12.2. Restructuring the social environment
12.3. Avoidance/reducing exposure to cues for the behavior
13.2. Framing/reframing
15.2. Mental rehearsal of successful performance
15.3. Focus on past success

bold = main BCT, BCTs are numbered according to Taxonomy V1

In addition to the theoretical underpinning of the intervention, the aim was also to allow for individual tailoring of the intervention content. The implementers of the intervention, i.e. coaches, were provided with questionnaire data from the participants before the first session, in which they gained motivational and volitional information. Tailoring continued throughout all sessions, in which the coaches were instructed to select and apply BCTs from the “Anchor List”, which fitted the assessed needs.

The coaches were sport science and psychology graduates. They were recruited in stages and trained by the study team. The training (duration of 8 weeks with 2 hourly sessions weekly) contained insight into the trial procedures including background on depression and behavior change in this population, underpinning theory and tailoring of the intervention, structure of the intervention including delivery mode, and the role of a coach including conversational conduct and relationship building. Additionally, the training included weekly exercises, listening to and analyzing audio recordings and a know-how-check, in which the coaches role-played a scenario with a member of the study team. The coaches received a manual containing the training content as well as a detailed guide of every session, including checklists and instructions for debriefing. The coaches were instructed on how to document the implementation of the intervention including number, duration and content of each session. For the remote phase of the intervention, a protocol was developed specifying the procedures in case of missed appointments, participants who were difficult to reach and defining the expected availability of the coaches. The implementation was monitored continuously by the study team during monthly team meetings with the coaches. Information regarding the intervention development can also be found in the study protocol [11].

### Data collection

#### Reach of the intervention

Data representing the reach of the intervention were demographic and clinical data, collected during in-patient treatment at screening. The clinician referring the participant to the study team communicated age, sex, physical activity level and depression diagnosis as well as depression severity to the study team confidentially. Depression diagnoses were stated according to ICD-10. Depression severity was assessed with the Beck Depression Inventory (BDI), containing 21 questions pertaining to depression symptoms resulting in a sum score ranging from 0 to 63 points [21]. Physical activity was assessed using the International Physical Activity Questionnaire (IPAQ), containing seven questions to elicit the amount of moderate and vigorous physical activity performed in the preceding seven days [22].

#### Dose and fidelity of the intervention

Data representing the dose and content of the intervention were collected by the coaches using a predefined tool, containing the date of contact with the participant, duration of preparation, counseling and debriefing and the content (MoVo as well as BCTs) of each counseling session. Additionally, the dates of all text messages sent were documented and for each counseling session there was the opportunity for free text comments. The coaches entered these data regularly after every counseling session and were monitored by the study team.

#### Adaptation to the intervention

Adaptations to the intervention were documented during the regular team meetings and the counseling materials were adjusted accordingly.

#### Satisfaction with the intervention

Satisfaction data were collected from the participants via questionnaire at two time points during their study participation. The first time was 6 weeks (post) and the second 12 months (follow-up) after discharge from in-patient treatment. The questionnaire contained questions regarding expectations, understandability of the content, satisfaction with the coach, helpfulness of the text messages, user friendliness and helpfulness of the mobile application, achieving (intermittent) goals, motivation to continue and recommendation of the intervention, suitability for health promotion, general satisfaction, and perceived effort in relation to success. These questions were all answered on a 4-point Likert scale ranging from no, mostly no, mostly yes to yes. The scale for the last question ranged from too high, mostly too high, appropriate to low. Additionally, there was a question pertaining to the perception of intervals between sessions, which was answered on a scale ranging from too long, just right to too short. Lastly, the question was asked whether a different fitness application was being used. This question was answered with yes or no.

### Data analysis

To analyze whether the duration and content of the counseling sessions differed according to the intervention dose, subgroups were defined according to intervention attendance. “Early dropouts” dropped out of the intervention after leaving in-patient treatment, “low dose” was defined as less than 75% of the remote intervention and “high dose” was defined as 75% or more of the intended remote intervention. This cut off is supported by meta-analytic data on the adherence to physical activity interventions in other chronic conditions (cancer, cardiovascular disease and diabetes), which show that the average adherence rate is 77% (95% CI 0.68 to 0.84) of the intended dose [23]. Additionally, number and duration of counseling sessions were separately analyzed for in-person and telephone counselling.

Metric data were reported in means (*M*) and standard deviations (*SD*). Group differences for metric data were analyzed with Analyses of Variance (ANOVA) tests and reported with *F*-statistics and eta-squared (η^2^). For comparisons of more than two groups Bonferroni post hoc tests were conducted. Group differences for categorical data were analyzed with chi-squared tests (χ^2^) and reported with corresponding chi-squared values and Cramer’s *V*, appropriate for contingency tables larger than 2 × 2. The significance level for analyses was set at *p* < 0.05 across all analyses. Analyses were performed in STATA (StataCorp. 2017. *Stata Statistical Software: Release 15*. College Station, TX: StataCorp LLC.).

## Results

### Reach

The intervention did reach the targeted audience and no significant differences were found between the groups of intervention attendance with regard to age, sex, depression severity and diagnoses, or study site. A total of 127 participants were recruited and randomized to the intervention group. By January 2022, 101 participants (80%) completed their participation in the PACINPAT trial, which comprises the current sample size. For the current analyses, three people were excluded because their primary diagnosis did not fit the defined inclusion criteria. Additionally, those who were randomized to the intervention group but did not participate in the intervention at all (*n* = 3) were excluded. This resulted in a total sample of *N* = 95 participants, whose characteristics are described in Table 3.

**Table 3:** Participant characteristics

	Total (***N*** = 95)	Early Dropout (***n*** = 18)	Low Dose (***n*** = 37)	High Dose (***n*** = 40)	ANOVA		
					*F*	η^**2**^	
**Age** in years, mean (*SD*)	42 (13)	42 (13)	42 (14)	43 (12)	0.18	0.00	
**Depression severity according to BDI**, mean (*SD*)	21 (12)	24 (12)	21 (11)	18 (12)	1.58	0.03	
					***χ***^***2***^	***V***	***p***
**Sex**, *n* (%)							
Women	50 (53)	11 (61)	20 (54)	19 (47)	0.97	0.10	0.615
Men	45 (47)	7 (39)	17 (46)	21 (52)			
**Depression diagnosis according to ICD-10,***n* (%)							
Moderate episode (F32.1)	24 (25)	6 (33)	10 (27)	8 (20)	2.58	0.12	0.859
Severe episode (F32.2)	14 (15)	2 (11)	4 (11)	8 (20)			
Recurrent depression, current moderate episode (F33.1)	35 (37)	6 (33)	15 (40)	14 (35)			
Recurrent depression, current severe episode (F33.2)	22 (23)	4 (22)	8 (22)	10 (25)			
**Study site**, *n* (%)							
Basel	20 (21)	4 (22)	12 (32)	4 (10)	6.27	0.18	0.393
Riehen	22 (23)	4 (22)	8 (22)	10 (25)			
Solothurn	18 (19)	4 (22)	6 (16)	8 (20)			
Münchenbuchsee	35 (37)	6 (33)	11 (30)	18 (45)			

*BDI* Beck Depression Inventory, *ICD-10* International Classification of Disease, 10^th^ Edition

### Dose

The planned number of counseling sessions was two in-person sessions during in-patient treatment and 26 telephone sessions with an interval of 14 days during 12 months.

**Fig. 2:**
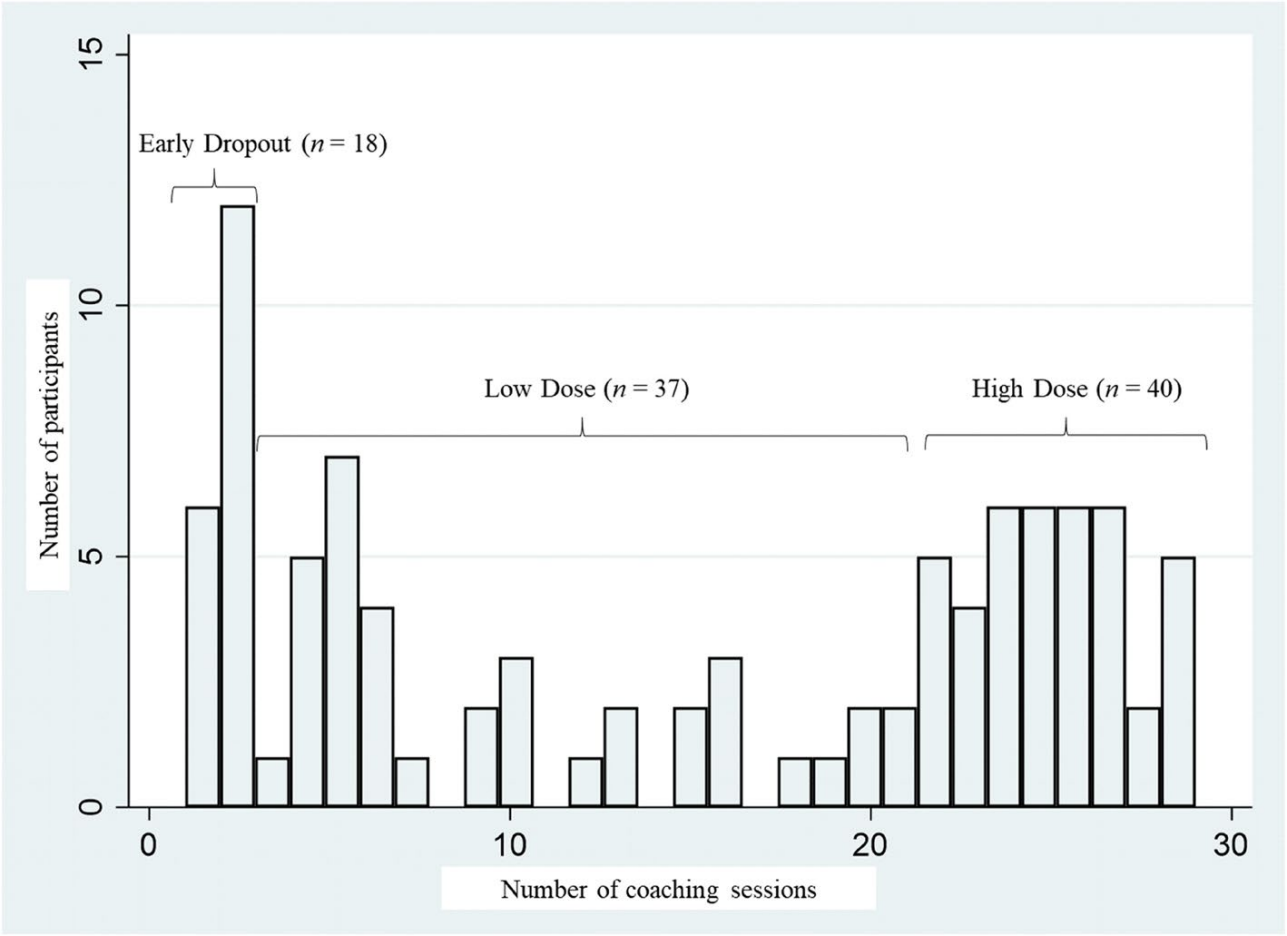
Intervention attendance

Not all participants achieved the planned number of counseling sessions. Hence, subgroups were created according to intervention attendance, which can be seen in Fig. 2.

The actual number of counseling sessions and their durations, in total as well as in the two intervention phases (in-person and telephone), are shown in Table 4. ANOVA tests revealed significant differences in all parameters referring to the number of sessions, thus confirming the three different groups of attendance. According to Bonferroni post hoc testing, only the number of in-person counseling sessions were not significantly different between the low and high dose groups.

**Table 4:** Intervention dose

	Total (*N* = 95)	Early Dropout (*n* = 18)	Low Dose (*n* = 37)	High Dose (*n* = 40)	ANOVA	
	***M (SD)***	***M (SD)***	***M (SD)***	***M (SD)***	***F***	***η***^***2***^
**Number of**						
Total counseling sessions	14.91 (10.24)	1.67^A,B^ (0.48)	10.05^B,C^ (5.88)	25.37^A,C^ (2.20)	268.79	0.85*
In-person	1.67 (0.48)	1.83^A^ (0.38)	2^A^ (0)	2^A^ (0)	18.64	0.29*
Remote	12.98 (10.15)	0^A,B^ (0)	8.05^B,C^ (5.88)	23.37^A,C^ (2.20)	264.50	0.85*
Messages	26.04 (20.81)	0^A,B^ (0)	16.65^B,C^ (14.43)	46.45^A,C^ (5.22)	172.62	0.79*
**Duration of preparation**						
In-person (min)	9.00 (9.46)	6.50 (5.21)	9.38 (8.41)	9.77 (11.61)	1.25	0.03
Remote (min)	2.63 (2.31)	–-	1.69 (1.55)	4.69 (1.49)	86.55	0.65*
**Duration of counseling**						
In-person (min)	57.37 (17.05)	45.39^A,B^ (20.38)	60.57^A^ (15.45)	59.80^B^ (14.74)	6.10	0.11*
Remote (min)	9.27 (8.34)	–-	5.44 (3.37)	16.99 (6.53)	99.23	0.68*
**Duration of debriefing**						
In-person (min)	9.09 (6.76)	5.55^A^ (3.73)	9.36 (6.98)	10.44^A^ (7.16)	3.46	0.07*
Remote (min)	3.77 (3.40)	–-	2.24 (1.79)	6.89 (2.50)	93.73	0.67*

*min* minutes. Capital superscript letters above the mean (*M*) indicate which of the three groups differed from each other based on Bonferroni post-hoc tests. Means (*M*) with the same letters are significantly different at *p* < 0.05

When considering the duration of the in-person counseling sessions, it is noteworthy that the “early dropout” group had significantly shorter sessions compared to both other groups. Correspondingly, the debriefing time for in-person counseling session was less. When considering the duration of the telephone counseling sessions, the “high dose” group had significantly longer preparation, counseling and debriefing time compared to the “low dose” group. With regard to intervals and duration of the remote intervention, the groups differed significantly. Intervals between the telephone counseling sessions differed significantly between the “high dose” (*M* = 17 days, *SD* = 2 days) and “low dose” (*M* = 25 days, *SD* = 10 days) groups (*F* = 99.75_(2,92)_, *η*^***2***^ = 0.68, *p* < 0.001). The overall duration of the remote intervention also differed significantly between the “high dose” (*M* = 13 months, *SD* = 1 month) and “low dose” (*M* = 6 months, *SD* = 5 months) groups (*F* = 107.27_(2,92)_, *η*^***2***^ = 0.70, *p* < 0.001).

### Fidelity

Fidelity, i.e. whether the content of the intervention was delivered as intended, was assessed by considering the content delivery as reported by the coaches and satisfaction as reported by the participants.

In Table 5 the content according to the MoVo Model is exemplified. Group differences already appeared in the second counseling session, in which plans were made less often in the “early dropout” group compared to both other groups. Additionally, the mobile application was introduced less frequently in the “low dose” group. Over half of the “early dropout” group discussed barriers (64%) and strategies (59%) even though this was intended in the third session (first telephone session).

**Table 5:** Content based on the motivation-volition model

	Intended counseling session	Total (*N* = 95)	Early Dropout (*n* = 18)	Low Dose (*n* = 37)	High Dose (*n* = 40)			
		***n* (%)**	***n* (%)**	***n* (%)**	***n* (%)**	**χ**^**2**^	***V***	***p***
Idea set	1	92 (97)	17 (94)	36 (97)	39 (97)	0.42	0.07	0.811
Goal set	1	91 (96)	17 (94)	36 (97)	38 (95)	0.35	0.06	0.839
Plan made	2	71 (75)	6 (33)	30 (81)	35 (87)	20.58	0.46	< 0.001
App explained	2	70 (74)	7 (39)	30 (81)	33 (82)	13.89	0.38	< 0.001
Barriers identified	3	61 (64)	13 (72)	20 (54)	28 (70)	2.75	0.17	0.253
Strategies identified	3	56 (59)	10 (55)	20 (54)	26 (65)	1.06	0.10	0.589
Intermittent goals		65 (68)	0	27 (73)	38 (95)	52.43	0.74	< 0.001
Overall goal reached	28	15 (16)	0	2 (5)	13 (32)	14.78	0.39	< 0.001

**Fig. 3:**
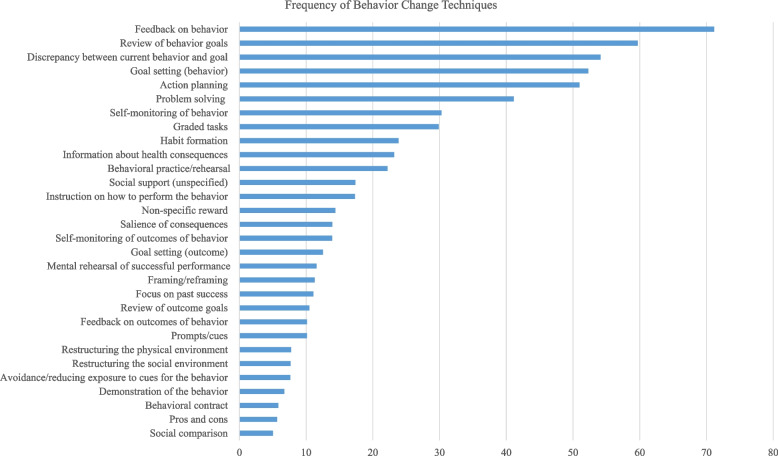
Frequency of Behavior Change Techniques

In Table 6 the content based on BCTs used during the telephone counseling is shown. Additionally, BCTs used in order of frequency can be seen in Fig. 3. According to ANOVA analyses, eleven of the BCTs were used significantly more frequently in the “low dose” group compared to the “high dose” group, three of which were main BCTs (self-monitoring of behavior (2.3.), social support (3.1.), information about health consequences (5.1.)).

**Table 6:** Content based on behavior change techniques

	BCT used at least once (*n* = 77)	Frequency of use relative to number of counseling sessions for at least one use (*n* = 77)			ANOVA		
	***N* (%)**	Total** (%)**	Low Dose** (%)**	High Dose** (%)**	***F***	**η**^**2**^	***p***
**1.1. Goal setting (behavior)**	71 (92)	52	58	48	2.50	0.03	0.12
1.3. Goal setting (outcome)	36 (47)	12	20	9	9.63	0.22	< 0.01
**1.2. Problem solving**	73 (95)	41	45	38	2.02	0.03	0.16
**1.4. Action planning**	74 (96)	51	51	51	0.01	< 0.01	0.93
8.7. Graded tasks	60 (78)	30	37	25	8.50	0.13	< 0.01
**1.5. Review of behavior goals**	68 (88)	60	62	58	0.41	< 0.01	0.52
1.7. Review of outcome goals	50 (65)	10	18	8	30.56	0.39	< 0.01
1.6. Discrepancy between current behavior and goal	35 (45)	54	50	57	0.26	< 0.01	0.61
1.8. Behavioral contract	7 (9)	6	8	5	0.39	< 0.01	0.56
**2.2. Feedback on behavior**	71 (92)	71	70	72	0.14	< 0.01	0.71
2.7. Feedback on outcomes of behavior	39 (51)	10	15	8	5.66	0.13	0.02
**2.3. Self-monitoring of behavior**	60 (78)	30	40	24	7.12	0.11	< 0.01
2.4. Self-monitoring of outcomes of behavior	11 (14)	14	24	10	5.03	0.36	0.05
**3.1. Social support (unspecified)**	44 (57)	17	27	12	11.75	0.22	< 0.01
**4.1. Instruction on how to perform the behavior**	48 (62)	17	26	13	11.45	0.20	< 0.01
**5.1. Information about health consequences**	62 (80)	23	33	16	15.63	0.21	< 0.01
5.2. Salience of consequences	39 (51)	14	16	13	1.56	0.04	0.22
6.1. Demonstration of the behavior	12 (15)	7	8	6	0.26	0.02	0.62
6.2. Social comparison	8 (10)	5	5	5	0.18	0.03	0.69
7.1. Prompts/cues	23 (30)	10	21	6	8.55	0.29	< 0.01
8.1. Behavioral practice/rehearsal	59 (77)	22	27	19	3.50	0.06	0.07
**8.3. Habit formation**	60 (78)	24	28	21	2.18	0.04	0.14
9.2. Pros and cons	11 (14)	6	5	6	0.02	< 0.01	0.90
10.3. Non-specific reward	30 (39)	14	19	11	3.71	0.12	0.06
12.1. Restructuring the physical environment	19 (25)	8	9	7	0.21	0.01	0.65
12.2. Restructuring the social environment	5 (6)	8	12	6	3.82	0.56	0.14
12.3. Avoidance/reducing exposure to cues for the behavior	18 (23)	8	15	6	7.65	0.32	0.01
13.2. Framing/reframing	22 (28)	11	13	10	0.76	0.04	0.39
15.2. Mental rehearsal of successful performance	27 (35)	11	16	10	4.12	0.14	0.05
15.3. Focus on past success	37 (48)	11	16	8	7.43	0.17	0.01

bold = main BCT, *BCT* Behavior Change Technique, BCTs are numbered according to Taxonomy V1

When comparing the ten most frequently used BCTs to the ten main BCTs on the “Anchor List”, discrepancy between current behavior and goal (1.6.) (54%) and graded tasks (8.7.) (30%) were among the top ten, however they were not among the main BCTs on the “Anchor List”. While the BCTs social support (3.1.) (17%) and instruction on how to perform behavior (4.1.) (17%) were among the main BCTs on the “Anchor List”, they were not among the ten most frequently used BCTs.

Results from the satisfaction questionnaire can be seen in Table 7. When considering the elements from the implementation evaluation dose and content, at post assessment, 74% of the participants rated the intervals between the counseling sessions (intended interval: 2 weeks), as too short, while at follow up assessment only 12% reported the intervals to be too short and 80% experienced them as just right. When considering intervention content, 76% (post assessment) and 80% (follow up assessment) reported the content to be appropriate and understandable. When considering other elements of the remote intervention, the text messages were reported to be helpful by approximately 70% of the participants at both assessment time points. The only area where lower rates of satisfaction were reported was pertaining to the mobile application (helping to stay motivated, to implement plans and reach goals). Approximately 30% of the participants rated these questions with “no” and a further 30% with “mostly no”. Overall, the participants reported that they were satisfied with their coach (89% post assessment, 86% follow up assessment).

**Table 7:** Participant satisfaction

	Post in-patient treatment (*n* = 63)				Post intervention (*n* = 50)			
	*n* (%)	*n* (%)	*n* (%)	*n* (%)	*n* (%)	*n* (%)	*n* (%)	*n* (%)
	**No**	**Mostly no**	**Mostly yes**	**Yes**	**No**	**Mostly No**	**Mostly yes**	**Yes**
Have your expectations been met?	3 (5)	7 (11)	22 (35)	31 (49)	4 (8)	6 (12)	10 (20)	30 (60)
Is the content appropriate and understandable?	0 (0)	0 (0)	15 (24)	48 (76)	0 (0)	0 (0)	10 (20)	40 (80)
Are you satisfied with your coach?	1 (2)	0 (0)	6 (9)	56 (89)	0 (0)	1 (2)	6 (12)	43 (86)
Would you prefer a different coach?	61 (97)	2 (3)	0 (0)	0 (0)	47 (94)	3 (6)	0 (0)	0 (0)
Are the weekly messages helpful to stay motivated?	6 (10)^a^	10 (16)^a^	21 (34)^a^	25 (40)^a^	3 (6)	5 (10)	20 (40)	22 (44)
Are the weekly messages helpful to implement your plans?	7 (11)	11 (18)	24 (38)	21 (33)	4 (8)^b^	7 (14)^b^	21 (43)^b^	17 (35)^b^
Are the weekly messages helpful to reach your goals?	8 (13)^a^	10 (16)^a^	23 (37)^a^	21 (34)^a^	3 (6)^b^	8 (16)^b^	22 (45)^b^	16 (33)^b^
Is the app user-friendly/understandable?					6 (12)^c^	8 (17)^c^	21 (44)^c^	13 (27)^c^
Is the App helpful to stay motivated?					15 (31)^b^	13 (26)^b^	16 (33)^b^	5 (10)^b^
Is the App helpful to implement your plans?					15 (31)^b^	12 (24)^b^	19 (39)^b^	3 (6)^b^
Is the App helpful to reach your goals?					16 (33)^b^	14 (28)^b^	14 (28)^b^	5 (10)^b^
Have you achieved intermittent goals that you set during the program?					3 (6)^b^	6 (12)^b^	27 (55)^b^	13 (27)^b^
Do you move more (increased duration or frequency)?	2 (3)	9 (14)	32 (51)	20 (32)	5 (10)	8 (16)	20 (40)	17 (34)
Are you motivated to continue your participation in the program?	1 (2)	3 (5)	24 (38)	35 (55)				
Would you recommend the program?	2 (3)	18 (28)	1 (2)	42 (67)	1 (2)^b^	3 (6)^b^	14 (29)^b^	31 (63)^b^
Do you think the program is a suitable health promotion measure?	1 (2)	0 (0)	19 (30)	43 (68)	0 (0)	0 (0)	21 (42)	29 (58)
Generally, are you satisfied with the program?	1 (2)	0 (0)	15 (24)	47 (74)	0 (0)	3 (6)	15 (30)	32 (64)
	**Too** **high**	**Mostly too high**	**Appro-priate**	**Low**	**Too** **high**	**Mostly too high**	**Appro-priate**	**Low**
How do you perceive the effort you put into participating in the program in relation to your success?	2 (3)	4 (6)	52 (83)	5 (8)	1 (2)	4 (8)	41 (82)	4 (8)
	**Too** **long**	**Just** **right**	**Too** **short**		**Too** **long**	**Just** **right**	**Too** **short**	
How do you perceive the intervals between the coaching sessions?	1 (2)	15 (24)	47 (74)		4 (8)	40 (80)	6 (12)	
	**No**		**Yes**		**No**		**Yes**	
Do you use a different Fitness App or activity tracker	48 (77)^a^		14 (23)^a^		36 (72)		14 (28)	

^a^Sample size of 62. ^b^Sample size of 49.^c^Sample size of 48

### Adaptation

Adaptations to the original MoVo intervention by Fuchs and colleagues [17] were made a priori to fit the PACINPAT context as stated in the study protocol [11]. The original design consists of three in-person sessions, the first and last as individual sessions and the second a group session. Given the in-patient structure at the study sites, group sessions were replaced by individual sessions. The third counseling session was conducted during a telephone counseling session as it could be expected that engagement would be greater in a remote setting versus an in-person setting once the participant had left in-patient treatment.

During the first weeks of the intervention it was decided that the timing of the MoVo content could be adapted by the coach if it became evident that the participant was not ready yet to discuss health goals, physical activity ideas and plans. In such cases, the initial counseling sessions were primarily used to build a working relationship. Additionally, during the remote intervention phase, the intervals between telephone sessions was adapted to the preference of the participant, however, a minimum of 2 weeks was maintained. These adaptations were carried out with all participants.

## Discussion

### Main results

This study gives a differentiated insight in to the implementation of the planned complex intervention, according to the MRC framework, including reach, dose, fidelity and adaption. With regard to reach, the PACINPAT intervention reached all but three participants in the intervention group. With regard to dose, the subgroup analysis showed that the “early dropout” group had significantly shorter in-person counseling sessions compared with the “low” and “high dose” groups. Additionally, the “high dose” group had significantly longer remote counseling sessions compared with the “low dose” group. With regard to fidelity, during in-person counseling sessions, physical activity plans were made less frequently and the mobile application was introduced less frequently in the “early dropout” group compared with the “low” and “high dose” groups. During remote counseling sessions, BCTs used in over 50% of the sessions were action planning (1.4.), goal setting (behavior) (1.1.), discrepancy between current behavior and goal (1.6.), review of behavior goals (1.5.) and feedback on behavior (2.2.). All but two of the BCTs on the “Anchor List” for physical activity were frequently used (social support [3.1.] and instruction on how to perform behavior [4.1.]). With regard to adaptation, the MoVo intervention was adapted in delivery mode and timing of content. The interval between telephone counseling sessions was adapted to the participants.

#### Intervention dose

Adherence to physical activity interventions in general may be reduced in people with major depression. According to a meta-analysis of RCTs, a dropout rate of 17.2% (95% CI 13.5 to 21.7) is to be expected [24]. With this in mind, 19% of the current sample dropping out of the physical activity intervention early is in keeping with expectations.

When considering adherence to the MoVo intervention specifically, in a study on the efficacy of the MoVo intervention in orthopedic patients, 90.1% of the intervention group received the intervention as intended [25]. Keeping in mind that the intervention duration consisted of three counseling sessions. Hence, this is comparable to the intervention dose, that even the “early dropouts” in the current study achieved. Additionally, approximately 35% of the orthopedic patients dropped out of the study, i.e. did not complete all assessments [25]. In a more recent study in which cardiac patients received the MoVo intervention, the implementation rate was not reported, however similarly a dropout rate from trial participation of approximately 37% was reported [26].

Adherence to physical activity programs may be linked with associations with physical activity, as shown by Antoniewicz and Brand [27]. In a dropout analysis of a 3-month physical activity program, they discovered that automatic positive associations towards exercising was a strong discriminating factor when investigating adherence patterns [27]. Implicit associations have been positively associated with physical activity behavior [28], however this has yet to be established in people with psychiatric disorders [29].

#### Intervention fidelity

During the in-person counseling sessions the “early dropout” group made plans less frequently compared with the “low” and “high dose” groups, even though no group differences were expected at that point. There is evidence suggesting that more severe depressive symptoms are associated with impaired volitional capacities resulting in reduced planning, and maintenance self-efficacy as well as higher susceptibility to distraction [30]. This may be a way of explaining the shorter counseling sessions as well as the differences in intervention fidelity. Nevertheless, with the present data, it is not possible to recognize whether the participants were less interested in participation or the coaches engaged them less in the intervention, resulting in the shorter duration.

In the remote counseling sessions, the frequently used BCTs in the intervention are in line with a recent remote intervention to promote physical activity in people with depression [31]. Noticeably, the BCTs used in this study pertain more strongly to the behavior than to the outcome (e.g., goal setting (behavior) (1.1.) is more frequently used than goal setting (outcome) (1.3.)). This reiterates that the content was implemented as designed, because these BCTs were defined as main BCTs. Additionally, given the cyclical nature of depression [32], it may be more attractive to focus on behavior, which is a more immediate concept, than on outcomes, which may be too far in the future and less within the sense of behavioral control [33].

There was a discrepancy between pre-defined main BCTs on the “Anchor List” and BCTS, which were actually frequently used. For example, the third most frequently used BCT, not among the main BCTs on the “Anchor List”, discrepancy between current behavior and goal (1.6.), may bridge the aforementioned gap between behavior and outcome that is needed.

The eighth most frequently used BCT, not among the main BCTs on the “Anchor List”, was graded tasks (8.7.). Again this reiterates that the content was implemented as designed. The coaches were instructed to encourage the participant to set an intermittent goal every two months, in an attempt to increase the participants’ belief in their capabilities over the duration of the intervention [20].

Conversely, there were some pre-defined main BCTs on the “Anchor List”, which were not frequently used. First, social support (3.1.), which includes the encouragement of engaging a “buddy”, housemate or partner in events or raising awareness for group activities. From the current analysis it is not possible to gauge whether the coaches did not use this BCT frequently because sufficient social support was provided or because the opposite was true [34]. In which case, in a remote setting, it was not possible for the coach to offer any further means of social support. Second, instruction on how to perform behavior (4.1.), which includes skill training and is related to behavioral practice and demonstration of the behavior. Despite the fact that remote interventions are an attractive option from the point of view of low cost and wide reach [35], they may not be the ideal platform to deliver BCTs of this nature. Alternatively, the chosen physical activity behaviors may not have been particularly complex and thus not requiring much instruction.

The remote counseling further included text messages and the use of mobile application. Evaluations of applications for mental health have found that most frequent persuasive techniques used are self-monitoring, personalization and reminder [36], which are in line with the concept of the PACINPAT remote intervention. According to the satisfaction data, the text messages were overall considered helpful. This was also the case in the preceding study of physical activity counseling in health physically inactive adults, however outcome analyses suggested that the text messages did not have an additional impact on actual physical activity behavior [9].

#### Intervention adaptation

The adaptations made to the MoVo intervention mean it was not fully implemented as intended in this population. Even though goals and ideas were set by most participants in the first session, planning, barriers and strategies were not discussed as frequently as expected. The MoVo model, as designed by Fuchs and colleagues [17], was intended for orthopedic patients. It has been implemented in people with obesity [37] with increased physical activity outcomes, however there is no information whether the intervention was implemented as intended. Currently, studies implementing the interventions based on the MoVo model in psychiatric [38] and oncology [39] populations are ongoing. It could be possible that the MoVo content is suitable for people with major depression, however there may need to be initial counseling sessions devoted to trust and relationship building before the behavior change content is addressed. This is supported by qualitative findings, iterating the importance of the source of physical activity support in people with severe mental illness [40].

Finally, the intervals of the telephone counseling sessions were adapted to the preferences of the participants. It proved challenging for the coaches to reach some of the participants in the intended interval of two weeks. It could be that, as with physical activity itself, the intervention may best be provided in the dose that suits the participant [41]. However, increased intervals between counseling sessions also result in longer breaks and in this case a shorter overall intervention duration. This in turn, assuming the counseling has an effect on behavior, has the potential of disturbing the repetition of the behavior in recurring contexts, leading to habit formation [42]. The implications of variability in intervals may become clearer in future outcome analyses.

### Implications

These results will help to interpret the outcome analyses of the PACINPAT trial. In particular, understanding different adherence groups and differences in dose of the intervention may facilitate the understanding of the behavioral outcomes [43].

The next step after an efficacy trial, in this case the PACINPAT trial, is to refine and upscale the intervention to truly implement this type of physical activity counseling in the practice of psychiatric care [44]. Knowledge gained from this trial may facilitate the refinement of the intervention, particularly in terms of dose and content in the early stages to potentially limit the early dropout rate. In terms of remote counseling, this study provides frequently used BCT, which may be recommended in the design of remote physical activity counseling for patients with depression. According to the public health research progression model, replication studies would then be necessary before increasing the scale [45]. More generally, this study may encourage the movement towards conducting more extensive evaluations in similar settings.

### Future research

Adherence guidelines, as they exist for medication [46], may be required for behavioral counseling interventions. The attendance cut off rate of 75% used to make the subgroups was supported by meta-analytic data, however, to date there seems to be little consensus regarding the definition and measure of adherence in physical activity interventions [47]. Some researchers do suggest that it is common to compare high and low adherence to exercise programs, whereby the former refers to ≥ 70% adherence and the latter < 70% adherence, as measured, for example, with accelerometry [48]. However, this pertains to adherence to physical activity and not to physical activity counseling interventions. Hence, future research could focus on the dose–response of physical activity counseling in patients with major depression.

In future, the fidelity of the intervention could additionally be rated by using video recording to allow a more objective fidelity rating. Furthermore, adding qualitative data may be beneficial to better understand why there were differences particularly concerning counseling session duration and content fidelity. Hence, adapting a mixed methods research design could be a recommendation for future research.

For a more comprehensive analysis of the implementation and to better understand the use of technology in this intervention, the use of the mobile application, timing of the receipt and reading of the text messages would need to be evaluated in future.

## Strengths and limitations

The strengths of this study include, first, answering the call for a more holistic approach to trial evaluations [13]. In so doing, this study addresses recommendations stating that theory-based complementary process evaluations are needed to enhance the generalizability of quantitative trials [49]. Second, evaluating the implementation of an intervention delivered during 12 months, may be particularly poignant, because the potential for variation in dose, content and adaptation may arguably be greater. Third, multiple data sources were considered. Data provided by the implementers are particularly valuable because they were captured directly after each counseling session, thus they are less prone to recall bias. Additionally, they provide information on the content of an individually tailored counselling session for each participant. The presented participant satisfaction data can also be seen as a strength, despite the early dropouts not being represented, because they did not take part in the post and follow up assessments. Overall, these quantitative data allow an objective and reliable evaluation of the reach, dose and fidelity of the intervention.

Despite these strengths, there are limitations to be considered. First, there is a potential for recruitment bias, because only people who were interested in becoming more physically active took part in the trial, thus leaving it unknown how the intervention would have been implemented with a less motivated sample. Second, the variability of the intervention dose does not allow general conclusions regarding the content and adaptation of the intervention. However, examining only selected participants would not correspond to practice. Therefore, the subgroup analysis was deemed a suitable solution to evaluate the intervention implementation concisely while preserving variability. Third, there are some limitations in the quantitative data. For example, it cannot be gauged why the early dropouts had shorter in-person counseling sessions, why some people preferred longer intervals during the remote counseling sessions or why coaches used some BCTs more frequently than others.

## Conclusion

The PACINPAT intervention reached the intended participants and was attended by early dropouts and participants engaging in low or high doses. The high dose comprised 75% of the intended number of counseling sessions in approximately the intended interval between sessions. Group differences were already recognizable in the first two sessions according to session length and fidelity of content. This may represent a vulnerable stage of the intervention. Further research is needed to validate this and investigate potential improvement strategies. Frequently used behavior-focused (as opposed to outcome-focused) BCTs and adaptations made in dose (longer intervals) and content (MoVo) provide interesting insight into the potential needs of the population receiving this intervention for the first time. Overall, the intervention was rated positively by the participants. These findings are important for designing and implementing physical activity interventions in psychiatric care and for the future interpretation of the PACINPAT outcome evaluations.

## Data Availability

The data and materials will be made available by the corresponding author (Robyn Cody) upon request, without undue reservation.

## Abbreviations

PACINPAT: Physical activity counseling in in-patients with major depression
CI: Confidence interval
Min: Minutes
RCT: Randomized controlled trial
MRC: Medical research council
ICD-10: International classification of disease, 10^th^ edition
BDI: Beck depression inventory
MoVo: Motivation volition
BCW: Behavior change wheel
BCT: Behavior change technique
IPAQ: International physical activity questionnaire
M: Mean
SD: Standard deviation
ANOVA: Analysis of variance
